# Evidence-based genomic diagnosis characterized chromosomal and cryptic imbalances in 30 elderly patients with myelodysplastic syndrome and acute myeloid leukemia

**DOI:** 10.1186/1755-8166-4-3

**Published:** 2011-01-20

**Authors:** Renu Bajaj, Fang Xu, Bixia Xiang, Katherine Wilcox, Autumn J DiAdamo, Rachana Kumar, Alexandra Pietraszkiewicz, Stephanie Halene, Peining Li

**Affiliations:** 1 Molecular Cytogenetics Laboratory, Department of Genetics, Yale University School of Medicine, 333 Cedar Street, New Haven, CT, USA; 2 Section of Hematology, Department of Internal Medicine, Yale University School of Medicine, 333 Cedar Street, New Haven, CT, USA; 3 Cytogenetics Laboratory, Departments of Pathology, Anatomy and Cell Biology, Thomas Jefferson University Hospital, Philadelphia, PA, USA; 4 Department of Cytogenetics, City of Hope, Duarte, CA, USA

## Abstract

**Background:**

To evaluate the clinical validity of genome-wide oligonucleotide array comparative genomic hybridization (aCGH) for detecting somatic abnormalities, we have applied this genomic analysis to 30 cases (13 MDS and 17 AML) with clonal chromosomal abnormalities detected in more than 50% of analyzed metaphase cells.

**Results:**

The aCGH detected all numerical chromosomal gains and losses from the mainline clones and 113 copy number alterations (CNAs) ranging from 0.257 to 102.519 megabases (Mb). Clinically significant recurrent deletions of 5q (involving the *RPS14 *gene), 12p12.3 (*ETV6 *gene), 17p13 (*TP53 *gene), 17q11.2 (*NF1 *gene) and 20q, double minutes containing the *MYC *gene and segmental amplification involving the *MLL *gene were further characterized with defined breakpoints and gene contents. Genomic features of microdeletions at 17q11.2 were confirmed by FISH using targeted BAC clones. The aCGH also defined break points in a derivative chromosome 6, der(6)t(3;6)(q21.3;p22.2), and an isodicentric X chromosome. However, chromosomally observed sideline clonal abnormalities in five cases were not detected by aCGH.

**Conclusions:**

Our data indicated that an integrated cytogenomic analysis will be a better diagnostic scheme to delineate genomic contents of chromosomal and cryptic abnormalities in patients with MDS and AML. An evidence-based approach to interpret somatic genomic findings was proposed.

## Introduction

The identification of recurrent chromosomal abnormalities in various leukemias and the understanding of molecular defects and pathogenic mechanisms underlying these abnormalities have made cytogenetic analysis valuable in providing diagnostic and prognostic parameters for disease stratification and treatment evaluation [1]. With an average resolution of 6-10 megabases (Mb) on a 300-500 G-band level, conventional karyotyping has been the current standard for screening chromosomal abnormalities on metaphases from direct and cultured bone marrow (BM) and leukemic blood (LB) cells. This approach requires mitotic active cells and frequently encounters difficulties due to the low mitotic index and poor chromosome morphology of leukemic cells. Fluorescence in situ hybridization (FISH) tests using targeted probes to detect gene/locus-specific rearrangements have enhanced the analytical resolution to 300-800 kilobases (Kb) and extended conventional metaphase analysis into interphase cells. Current cytogenetic analysis for patients with myelodysplastic syndrome (MDS) and acute myeloid leukemia (AML) involves cell-based conventional chromosomal analysis and FISH assays using a panel of targeted probes [2,3]. We have previously validated a DNA-based genome-wide oligonucleotide array comparative genomic hybridization (aCGH) for clinical diagnosis of constitutional chromosomal abnormalities and genomic disorders in pediatric patients with mental retardation and developmental delay [4]. The clinical utility of this aCGH based on Agilent's 44K design (CGH4410B) has demonstrated an average analytical resolution of 300-500 Kb and an improved abnormal detection rate from 5-7% by conventional chromosome and FISH analyses to 12% by aCGH [5]. Evidence-based guidelines to interpret genomic findings in the pediatric patients have been proposed [6,7]. Recently, genome-wide analyses using BAC-clone aCGH, oligonucleotide aCGH and SNP array have been applied in a research or an exploratory setting to profile the genomic alterations in patients with MDS and AML [8-15]. To evaluate the diagnostic value of aCGH in detecting somatic chromosomal and segmental copy number alterations (CNAs), we have performed aCGH analysis on 30 MDS and AML cases with different clonal abnormalities. The results further characterized the genomic complexity of recurrent chromosomal deletions, duplications, amplifications and cryptic aberrations. Despite its inherent limitation in detecting recurrent balanced reciprocal translocations and low level secondary clonal abnormalities, the aCGH analysis provides detailed genomic features of simple and complex chromosomal abnormalities and cryptic aberrations otherwise not detectable by conventional G-band and FISH assays. Integrated chromosome and genomic analyses and evidence-based interpretation should be a standardized cytogenomic procedure for patients with MDS and AML.

## Materials and methods

### Patient Samples

The Yale cytogenetics laboratory is CLIA-approved and provides diagnostic services to patients with various hematopoietic disorders and solid tumors. Follow up aCGH analyses had been performed on 30 MDS (n = 13) and AML (n = 17) patients with clonal chromosomal abnormalities detected in > 50% of BM or LB cells. All except one (case #17) were elderly patients with ages ranging from 51 to 93 years (average 67 years, Table 1). The criteria regarding the technical feasibility and medical necessity for pursuing diagnostic aCGH was: 1) sufficient residual BM or LB sample available for DNA extraction and clonal chromosomal abnormality detected in > 50% of BM or LB cells analyzed by conventional cytogenetics, 2) presence of chromosomally unresolved complex rearrangement or marker chromosome of unknown origin, and 3) genomic aberrations suspected in addition to the age-related Y chromosome loss and other simple chromosomal abnormalities. Informed consent was obtained from patients for use of residual materials on further genomic diagnosis.

**Table 1:** Recognized chromosomal abnormalities in the 30 patients with MDS and AML

Case#	Age(yr)	Sample	Type	Chromosome/FISH Results*
1	71	BM	AML	45,XY,**del(5)(q11.1q35.1)**,-11,-12,add(17)(p11.2),i(22)(q10)add(q13),+3mar[13]

2	74	BM	MDS	46,X,t(X;3)(p21;p14),**del(5)(q21q33)**[20]

3	86	LC	MDS	50-55,XX,+1,**del(5)(q23q34)**,+9,+11,+13,+14,dup(22)(q11q13),+3mar[cp20]

4	73	BM	AML	44,XX,der(5)t(5;17)(q35;q12)**del(5)(q14q34)**,del(7)(p11.2),del(9)(p23p23),-17,-18,t(22;22)(q13.3q11.2)dup(22)(q11.2q12.3)[15]

5	77	LC	AML	42,XX,**del(5)(q12q33)**,-7,idic(8)(p12),dic(12:16)(p13;p13.3),-18,-20,-21,+mar[20]

6	68	BM	AML	45,XX,t(1;11)(p22;q22),del(2)(p13p23),del(4)(q11.2q13.3),**del(5)(q14q33)**,**del(7)(q22q36)**,-12,del(13)(q14q34)[14]

7	51	BM	MDS	46,XX,**del(5)(q14q33)**[5]/45,idem,dic(17;20)(p11.2;q11.2)[9]

8	53	BM	MDS	44,XX,del(4)(q13q28),**-5**,t(7;9)(q32;p13),del(12)(p11.2p13),der(17)t(5;17)(p11;p11)[18]

9	61	LC	MDS	44,XY,**-5**,der(7)t(7;12)(p22;q13),r(9),der(10)t(5;10)(p13;p15),add(11)(q23),-12,-13,add(21)(p11),+1-2mar[cp14]

10	55	LC	AML	46,XY,**t(6;6)(p23;q16)**[11]

11	63	LC	AML	45,XY,**-7**[19]

12	63	BM	MDS	46,XY,der(6)t(3;6)(q21.3;p22.2),**del(7)(q21.13q31.33)**[16]

13	78	BM	AML	46,XX,del(1)(q12),+del(1),der(2)t(2;3)(p21;p21),del(2)(q31q37),add(5)(q35),**del(7)(q22q36)**,trp(11)(q13q25),add(17)(q25),+mar[cp19]

14	63	LC	AML	47,XY,**+8**[20]

15	71	BM	MDS	46,XY,t(3;21)(q26;q22),**+8**[17]

16	93	LC	AML	50,X,-Y,+4,+5,+7,**+8,+8**[cp20]

17	20	LC	AML	44,XY,**der(8)t(8;17)(p11.2;q11.2)**,-17,-19,-21,+mar[cp20]

18	88	BM	MDS	46,XY,**del(9)(q12q31)**[20]

19	74	BM	MDS	47,XX,**del(9)(q13q31)**,+18,**4-50dmin**[20]

20	78	BM	MDS	47,XY,**+11**[18]

21	66	BM	AML	46,XY,**t(11;19)(q23;p13.1)**[14]/46,idem,del(9)(q21q32)[6]

22	60	BM	AML	50,XX,+der(1)t(1;13)(q10;q10),+6,+8,t(8:16)(q22;p13),**t(11;19)(q23;p13.1)**,+19,+20[20]

23	51	LC	AML	47,XX,**t(15;17)(q22;q21.1)**,+mar[20]

24	73	BM	MDS	46,XY,**der(17)t(9;17)(p21.1;q25.1)**[18]

25	63	BM	AML	46,XY,**del(20)(q11.2)**[16]

26	51	BM	MDS	46,XX,**del(20)(q11.2)**[10]/47,XX,+8[7]

27	79	BM	AML	47-48,X,**idic(X)(q13),+idic(X)**[10]/47,idem,+8[3]

28	61	BM	AML	45,X,**-Y**[20]

29	69	LC	AML	45,X,**-Y**[10]/47,XY,**+der(1)t(1;19)(p13;p13)**,t(16;20)(q21;q12)[10]

30	79	BM	MDS	45,X,**-Y**[17]/50,idem,+X,+15,+20,-22,+3mar[4]

* **bold **for most significant chromosomal abnormality in each case

### Conventional Karyotyping and FISH Testing

Conventional chromosome analysis was performed on submitted BM and LB specimens using our laboratory's standardized protocols. Routine FISH tests were performed using a MDS/AML panel of commercial probes for the 5q (*EGR1 *gene at 5q31), 7q (*D7S486 *at 7q31), 8q (*MYC *at 8q24) and 20q (*D20S108 *at 20q12) loci and for the *RUNX1T1 *(*ETO*, 8q21.3), *ETV6 *(*TEL*, 12p13.2), *RUNX1 *(*AML1*, 21q22), *MLL *(11q23.3), *PML *(15q22), *RARA *(17q21.1), *CBFB *(16q22) genes and other relevant loci (Abbott Molecular, Des Plaines, IL). To confirm significant cryptic genomic aberrations, DNA samples from two BAC clones, RP11-1107G21 (*NF1 *gene at 17q11.2, chr17:26,415,260-26,627,398, sequence designation per NCBI36/hg18 assembly of the UCSC Human Genome browser http://genome.ucsc.edu/ and RP11-55J8 (*RHOT1 *gene at 17q11.2, chr17:27,462,203-27,654,151), were purchased from Roswell Park Cancer Institute (Buffalo, NY). The labeling of BAC DNA with fluorescent nucleotides by nick translation, the hybridization and the image analysis were performed as previously described [4,16].

### The aCGH Analysis and Data Analysis

Genomic DNAs were extracted from the residual BM or LB specimens using Puregene Kit by following manufacturer's instruction (Qiagen Inc., Valencia, CA). DNA concentration was measured using a NanoDrop spectrophotometer (ND-1000, Thermo Fisher Scientific Inc., Waltham, MA) and high molecular weight DNA quality was verified by agarose gel electrophoresis. For each aCGH analysis, 2.5 ug of test genomic DNA from the patient and 2.5 ug of control DNA from a sex-matched or -mismatched healthy individual were used following the manufacturer's protocol for the Agilent Human Genome aCGH microarray 44K kit (Agilent Technologies Inc., Santa Clara, CA). This laboratory has validated the aCGH procedure to offer 99% sensitivity and 99% specificity with an average analytical resolution of 300-500 Kb using the log_2 _ratio from five to seven contiguous probes, and also demonstrated its capacity in detecting 25%, 33% and 50% level of mosaicism [4]. The differential labeling of test and control DNAs, comparative hybridization onto 4x44K Agilent slides, post-hybridization wash, slide scanning, image feature extraction were processed as previously described [4,16]. Data was analyzed using Agilent's DNA Analytical (version 4.0) with the built-in ADM-2 algorithm set at threshold value of 6, a cut off value of 0.25, and a filter of six probes. All CNAs except the recognized copy number variants from the Database of Genomic Variants http://projects.tcag.ca/variation/ were recorded. The base pair designations from the Agilent 44K array are according to the March 2006 Assembly (NCBI36/hg18) on the UCSC Human Genome browser http://genome.ucsc.edu/. The aCGH finding from each case was compared with the chromosomally detected clonal abnormality to further define the breakpoint and the gene content involved. Raw data from the 30 cases were loaded onto the Nexus5 Software (BioDiscovery, Los Angles) to evaluate the genome-wide distribution and relative frequency of chromosomal and genomic alterations.

## Results

**Figure 1:**
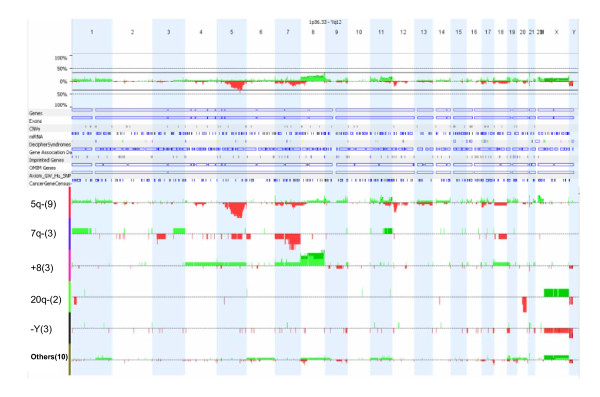
**Genome-wide incidence map of abnormal findings from the 30 MDS and AML cases**. Complied deletions (red) and duplications (green) from all cases are shown in the top panel and from subgroups based on the major chromosomal abnormalities of 5q deletion (n = 9), 7q deletion (n = 3), trisomy 8 (n = 3), 20q deletion (n = 2), loss of Y chromosome (n = 3) and others (n = 10) are shown in the bottom panel.

The detected clonal chromosomal abnormalities of the 30 patients were listed in Table 1. All recurrent chromosomal deletions and translocations were confirmed by FISH tests using targeted probes. The aCGH analysis detected all main clone numerical chromosomal abnormalities and chromosomally-observed segmental deletions and duplications. The observed numerical abnormalities included gains of chromosomes 1, 4, 5, 6, 7, 8, 9, 11, 13, 14, 18, 19, 20 and 22, and losses of chromosomes Y, 5, 7, 13 and 18. No genomic aberration was noted in two patients with a balanced translocation (cases #10 and #21, Table 1). In five cases (cases #21, #26-27, #29-30, Table 1), sideline clones with unbalanced chromosomal abnormalities were not detected by aCGH. A total of 113 CNAs with size ranging from 0.257 to 102.519 Mb were found in 23 patients (an average of 4.8 CNAs per case and of 20.241 Mb per CNA, see Additional file 1, Table S1). The genome-wide distribution of chromosomal and genomic copy number alterations was plotted using the Nexus5 software (Figure 1). Cases sharing similar cytogenetic abnormalities, such as the 5q deletion or monosomy 5 (n = 9), 7q deletion or monosomy 7 (n = 3), trisomy 8 (n = 3), 20q deletion (n = 2), loss of Y (n = 3) and other abnormalities (n = 10), were grouped together to estimate the frequency of other associated aberrations. It was noted that 5q deletions showed many associated recurrent aberrations, especially the 12p deletion (5/9 cases), 17p deletion (3/9) and 17q deletion (2/9). The size distribution and percentage of detected CNAs listed in Table 2 indicated that conventional chromosome analysis with a resolution of 5~10 Mb can miss a significant proportion (44%, 50 CNAs < 10 Mb out of 113 CNAs) of genomic aberrations.

**Table 2:** Size distribution of genomic imbalances

Size	No. of Imbalances	Percentage
< 1 Mb	5	3.5
1~5 Mb	30	20.8
5~10 Mb	15	10.4
10~20 Mb	23	16
> 20 Mb	40	27.8
Chr. Gain/Loss	31	21.5

Total	144	100

No. Imbalances per case	No. Of Cases	Percentage

0	2	6.7
1~5	19	63.3
5~10	4	13.3
> 10	5	16.7

**Figure 2:**
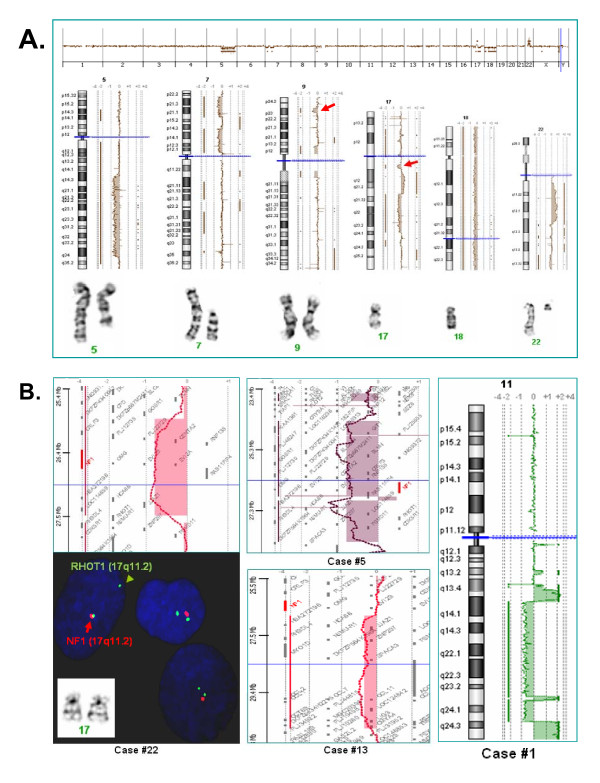
**Genomic features of chromosomal abnormalities and cryptic alterations**. A) Genome and chromosome views and correlated chromosomal rearrangements for case #4 show large deletion in 5q, truncated deletions in 7p, cryptic deletions in 9p23 and 17q11 (arrows), large duplication and deletion in 17q, a loss of a chromosome 18, segmental duplication and deletion in 22q. The 17q12-q21.31 duplication and 17q21.31-q24.1 deletion may be translocated onto the deleted 5q, and the 22q11.21-q12.3 duplication and 22q13.31-qter deletion may be initiated from a 22q/22q translocation. B) Left panel shows a cryptic genomic deletion of 17q11 in case #22 and FISH using probes for the *NF1 *and *RHOT1 *genes confirmed the *NF1 *gene deletion. Middle panel shows a deletion of 17q11.2 including the *NF1 *and *RHOT1 *genes in case #5 and a 17q11.2 deletion distal to the *NF1 *gene in case #13. Right panel shows complex 11q deletion and amplification involving the *MLL *gene at 11q23.3 in case #1.

The comparison between chromosomal abnormalities and genomic CNAs further delineated the breakpoints and gene contents involved in both simple and complex chromosomal rearrangements. For example, of the complex karyotype of case #4, the chromosomally observed 5q deletion was actually a result of an 80.5474 Mb deletion of 5q14.3-q34 followed by a fusion of structurally rearranged 17q (segmental deletions and duplication); the 7p deletion was a result of a 20.596 Mb deletion of 7p14.3-p11.2 and a 17.391 Mb deletion of 7p22.1-p15.3; the 9p had a 3.050 Mb microdeletion of 9p23; and what was denoted as additional material onto a 22q and a marker chromosome was most likely a result of a 22q/22q translocation with a 15.235 Mb duplication and a 11.694 Mb triplication of 22q11.2-q12.3 and a 5.199 Mb deletion of 22q13.31-q13.33 (Figure 2A, Additional file 1, Table S1). The case-by-case comparison of chromosomal and genomic findings not only defined the chromosomal abnormalities, but also demonstrated the genomic heterogeneity of recurrent chromosomal abnormalities. Chromosomally observed recurrent deletions of 5q, 7q, 9q, 12p, and 20q were all characterized by aCGH and confirmed in ten, three, two, six, and three cases, respectively. Of the 10 cases (cases #1~9 and #13 in Table 1) with a 5q deletion or monosomy 5, a simple deletion of various sizes was seen in seven cases and compound deletions of two or three segments were found in three cases (Additional file 1, Table S1). The deletion of the *ETV6 *gene at 12p13.2, a gene essential in hematopoiesis and frequently encountered in translocations in acute leukemias and MDS, was noted in five of the six cases with the 12p deletion, including in case #13 a microdeletion of 1.154 Mb confirmed by FISH using the *ETV6 *probe. Segmental rearrangements involving the *TP53 *gene by either the whole arm loss or a large deletion of 17p were noted in four cases (#1, #7-8 and #17). A chromosomally undetected deletion of 10.981 Mb at 9p21.3-p21.1 involving the *CDKN2A *and *CDKN2B *genes was noted in case #16. Chromosomal analysis recognized two unrelated clones in case #26 featuring a 20q deletion and trisomy 8, respectively. The aCGH detected a 14.920 Mb deletion of 1p36.21-p35.3 and a 20.202 Mb deletion of 20q11.23-q13.3 but not the unrelated clone with trisomy 8. This result suggested that the 1p deletion region may harbor haploinsufficient candidate genes relevant to the etiology or evolution of MDS and AML.

Microdeletions of 1.510 to 3.615 Mb encompassing the *NF1 *gene at 17q11.2 were found in three cases (cases #4, #5 and #22). FISH analysis using BAC clone probes for the *NF1 *and *RHOT1 *genes confirmed these deletions (Figure 2B). It was interesting to observe a transition pattern in case #4 with deletions of *NF1 *alone, *NF1 *and *RHOT1*, and *RHOT1 *alone in 11%, 12.5% and 34% of cells, respectively. In case #13, a deletion involving the *RHOT1 *gene but not the *NF1 *gene was noted and confirmed by FISH. These results suggested that the 17q11.2 region may contain hot spots for initiating deletions from either one or both proximal and distal orientations.

The dmin chromosomes observed in case #19 were further defined as a 4.277 Mb amplification of 8q24.13-q24.21 containing genes *TRIB1*, *MYC *and *CCDC26*. From the log2 ratio of 2.53 for this amplified region, it was estimated that there were average four dmin chromosomes in each leukemic cell. Compound deletions, triplications or quadruplications for 11q were noted in cases #1 and #13, which resulted in complex intrachromosomal rearrangements carrying amplified segments of the *MLL *gene (Figure 2B). The derivative chromosome 6, der(6)t(3;6)(q21.3;p22.2), in case #12 was further defined with breakpoints involving the *RAB43 *gene at 3q21.3 and the *KIAA0319 *gene at 6p22.2; and the isodicentric chromosome of Xp in case #27 was resulted from break and fusion distal to the *PHKA1 *gene at Xq13.1 (Additional file 1, Table S1).

## Discussion

Cytogenetics has played a major role in both diagnosis and prognosis in patients with hematological malignancies. The newly revised WHO classification of acute myeloid leukemias categorizes AML into subgroups with recurrent genetic abnormalities, with MDS features, treatment related and "not otherwise specified"; the last one is by far the largest category [1]. Cytogenetics is key components of both the international prognostic scoring (IPSS) and the WHO classification-based prognostic scoring systems (WPSS) for the myelodysplastic syndromes [17,18]. However, within all categories prognostic variation is observed, likely among others due to low sensitivity of conventional cytogenetics [12]. Our data demonstrated that aCGH can detect chromosomal, segmental and cryptic aberrations in LB and BM cells from MDS and AML patients. As summarized in Table 2, the aCGH detected an average of 4.8 CNAs per case ranging from 0 to 22, of which an approximately 44% were less than 10 Mb and not evident by conventional cytogenetic analysis. It is noteworthy while on one hand some relatively large deletions and duplications (> 10 Mb) were missed or unresolved by conventional chromosomal analysis, on the other hand chromosomally detected sideline clones were missed by aCGH. Therefore, an integrated cytogenomic approach using chromosome analysis, FISH assay and aCGH would definitely improve the analytical resolution and abnormality detection rate for MDS and AML patients.

**Figure 3:**
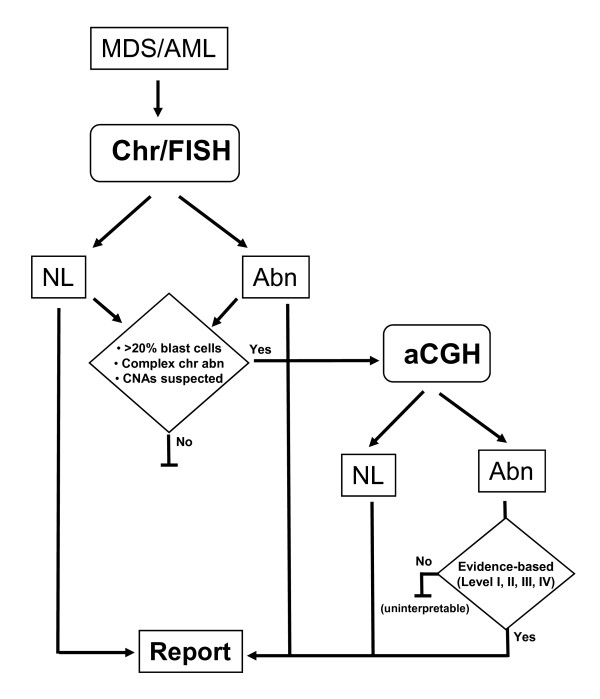
**Integrated cytogenomic workflow and proposed algorithm for interpreting and reporting clinically significant genomic findings**.

The interpretation of the clinical significance of somatic CNAs has been challenge especially for cases with numerous genomic aberrations. An evidence-based approach for interpreting constitutional genomic aberrations could be adopted for somatic CNAs with the classification of research evidence into four levels [6,7]. Level I evidence is derived from recognized disease-causing clonal chromosomal abnormalities in the WHO classification [1] or from well-designed systematic studies; level II evidence comes from series of diagnostic studies; level III evidence is derived from cohort design observational studies or basic research of gene functions and disease mechanisms on in-vitro systems and/or model animals, and level IV is based on case reports or expert's opinions. Genomic findings without evidence were documented as unknown significance and uninterpretable in the laboratory's database but not presented in the diagnostic report. We propose a workflow chart to illustrate the integration of chromosome, FISH and aCGH procedures and the algorithm for interpreting results (Figure 3).

Several systematic genomic studies of MDS and AML patients have revealed recurrent and hidden CNAs and provided level I or II evidence for diagnostic interpretation of genomic findings [8-15]. Rucker FG et al. [8] performed 2.8 K (2,799 BAC/PAC clones) aCGH analysis of 60 AML cases with complex karyotypes and found that the most frequent losses were of 5q (77%), 17p (55%) and 7q (45%) as well as the most frequent gains of 11q (40%) and 8q (38%). Suela J et al. [9] used the 44K oligonucleotide aCGH to study 100 consecutive de novo AML cases and noted most recurrent changes of losses of chromosomes 5, 7, 17 and 20 and gain of chromosome 8. Tyybakinoja et al.[10] used the same 44K oligonucleotide aCGH to analyze 26 AML patients with a normal karyotype and detected deletions of 8q24.3, 12p12.3, 1q41, 18q21.32 12p13.2 and duplications of 3p21.3 and 8q24 in four cases. Akagi T et al.[11] used SNP chip on 38 AML/MDS patients with a normal karyotype and detected CNAs of 3p, 5p, 5q, 7q, 11q, 17p and 17q as well as trisomies of chromosomes 8, 21 and 22. Starczynowski DT et al.[12] used 32K (32,433 BAC clones) tiling aCGH on CD34+ cells from 44 MDS patients and observed that frequent cryptic alterations included gains of 11q and 17q and losses at 2q and 5q. Walter MJ et al.[13] reported that, of 86 adult patients with de novo AML analyzed by SNP array, 40% of patients with an abnormal karyotype also had additional CNAs and 24% of patients with normal cytogenetics had CNAs. Interestingly, similar analysis performed on 111 children with de novo AML noted a low burden of genomic alterations [14]. A recent exploratory study on 30 cases of MDS, myeloproliferative neoplasia (MPN) or AML detected genomic aberrations in 24 cases with concordant aCGH/chromosome results in 83% of samples and new CNAs in 47% of cases; and normal aCGH results were noted in four cases with low percentage clonal abnormality (0.7~5%) detected by chromosome analysis [15]. The recurrent genomic alterations and some known genes within the alterations from these studies were summarized in Table 3. These studies and our data indicated that genomic analysis can detect CNAs in 15-40% of AML cases with a normal karyotype, in 40-90% of MDS/AML cases with abnormal clones and in almost 100% of cases with complex chromosomal findings.

**Table 3:** Representative studies showing evidence for relevant genomic alterations in MDS and AML

Patients	Case#	Methods	ADR*	Significance Genomic Findings	EL*	Refs
AML with complex karyotype	60	2.8K BAC/PAC aCGH	100%	Frequent losses of 5q(77%), 17p(55%), 7q(45%), 16q, 18q, 17q, 3p, 12q, 20q, 12p, 18p, 13q, 11q; gains/amplifications of 11q(40%), 8q(38%), 21q, 1p, 9p, 22q, 13q, 6p, 19p	II	8

de novo AML	100	44K oligo-aCGH (Agilent)	74%	Five smallest overlapping regions of imblances: 5q31.3, 16q23.1, 16q24.2, 17q11.2(NF1), 18p11.2	I	9

AML with normal karyotype	26	44K oligo-aCGH (Agilent)	15%	Cryptic losses of 8q24.11, 12p12.3, 1q41, 18q21,32, 12p13.2(ETV6), gain 3p21.3, dmin 8q24.13-q24.21(MYC)	II	10

MDS/AML with normal karyotype	38	SNP-chip (Affymetrix)	49%	CNN-LOH of 1p, 6p, 8q, 13q, 19p, 5q, 12q, 21q, 9p; losses of 17q11.2(NF1), 12p13.31p13.2(ETV6), 2q36.2, 4q24, 9p21.3p21.2(CDKN2A), 3p26.3, 14q21.2, 21q21.2, 8p23.2, 2p23.1; gains of 1q43, 18q21.2, 8q24.13q24.21(MYC)	II	11

Low risk MDS (CD34+ cells)	44	32K BAC tiling array	82%	Recurring common regions: losses of 2p23.1, 2q33.1-q33.2, 4p14, 5q13.1q13.2, 5q14.3q33, 5q33.3, 6p23, 10q21.3, 14q12, 19p12p13.2, 20q11.21q13.13, 22q13.1q13.2; gains of 7q34, 11q12.2, 11q24.2qter, 17q11.2, 17q12	II	12

Adult de novo AML	86	SNP 6.0 genechip (Affymetrix)	40%	12 recurring alterations found from 201 CNAs: losses of 3p14.1(FHIT), 5q31.1(CTNNA1), 7q31.31, 12p12.3(ETV6), 16q22.1(CEFB), 17p13.1(TP53), 17q11.2(NF1), 18p11.31; amplifications of 8q23.2(MYC), 11q23.3(MLL), 19q13.43, 21q22.2(ETS2)	I	13

Pediatric de novo AML	111	SNP-chips (Affymetrix)	Low#	Signficant losses of 5p15.33, 7p21.3, 7q36.1, 8q21.3 (RUNX1T1), 9p21.3 (CDKN2A), 9p21.2(TUSC1), 9p22.33(XPA), 11p14.1, 11q23.3(MLL), 12p13.31, 16p13.11(MYH11), 16q22.1(CBFB), 18p11.21; amplifications of 8q24.21(CCDC26), 13q32.1(ABCC4), 19p13.2), 21q22.2(ERG, TMPRSS2), 22q12.3.	I	14

MDS/MPN/AML with abnormal karyotype	30	SignatureChipWGBAC (v1.01)	80%	Cryptic RUNX1 deletions, hidden deletions of 3q26.2(EVI1), 5q22(APC),5q32(TCERG1), 12p13.1(EMP1), 12q21.3(KITLG), 17q11.2(NF1), gains of 12p13.32(CCND2)	II	15

MDS/AML with abnormal karyotype	30	44K oligo-aCGH (Agilent)	93%	Recurring alterations: losses of 5q(RPS14), 12p12.3(ETV6), 17p13(TP53), 17q11.2(NF1), 20q; gains of 8q24(MYC),11q23.2(MLL).	II	this report

*ADR: abnormality detection rate, EL. evidence level, #percentage not specified

Basic researches toward the understanding of gene functions and disease-causing mechanisms or case reports with unique clinical and genomic findings could provide level III and IV evidence for reporting genomic alterations. Detailed genomic analyses of the malignant cells in MDS and AML patients is likely to yield specific types or regions of recurrent chromosomal and genomic abnormalities providing further evidence for disease association and allowing identification of to date unknown candidate genes. Eventually, further classification of disease and targeted treatment can potentially result from such knowledge. The 5q deletion is possibly the best understood recurrent chromosomal deletion in myeloid malignancies. Two common deletion regions (CDR) have been identified conferring either a good (CDR1) or a poor (CDR2) prognosis, but usually changes are more complex [19]. All 5q deletions in our 10 cases and in another study of 12 MDS cases with an isolated 5q deletion by Evers C et al. [20] involved the loss of the *RPS14 *gene at 5q33.1, which support the causal role of *RPS14 *haploinsufficiency and were specified in the diagnostic reports [21]. Other haploinsufficiency genes or *miR-145 *and *miR146a *at 5q could also have causal or modifying effects [22-24], and should also be referred in the report. A der(6)t(3;6)(q21;p22) in a patient with AML at relapse and a t(1;6)(q21;p22) in another patient were reported [25]. We reported here a der(6)t(3;6)(q21.3;p22.2) likely caused by fusion of the *RAB43 *gene at 3q21.3 and the *KIAA0319 *gene at 6p22.2. Analysis of additional cases with similar 6p22 translocations could clarify if this is a recurrent primary or secondary rearrangement in AML. Monosomy 7 and 7q deletions portend a particularly poor prognosis in myeloid malignancies. The candidate genes within the recurrent 7q deletion are still under investigation and a recent study suggested that the *SAMD9*, *SAMD9L *and *Miki *(LOC253012) as the candidate genes for 7q21.3 [26]. Two out of the three cases with 7q deletions in our patients had the 7q21.3 deletion. As reported in the literature [16,27,28] and also shown from our cases #1, #13, # 19 and #23, genomic analysis has been used effectively to define dmin chromosomes of 8q24 and complex 11q rearrangements with or without the *MLL *gene amplification in simple or complex karyotype. The presence of cytogenetic unresolved marker chromosome in our case #23 and the reported hidden abnormalities associated with t(15;17) justified further genomic analysis for this obvious balanced rearrangements [29,30]. Microdeletions at 17q11.2 involving the *NF1 *gene are considered to be recurrent cryptic alterations in three of our cases (#4, #5 and #22) and also documented in several reports [9,11-13,31]. The presence of clustered flanking repetitive sequences of the *NF1 *locus is a likely explanation for recurrence of both constitutional and the somatic deletions [32]; further characterization of the noted transition pattern for the somatic 17q11.2 deletion could lead to better understanding of its mutagenesis mechanism.

In conclusion, our current diagnostic application of aCGH and accumulated evidence from previous studies support an integrated cytogenomic approach with evidence-based interpretation in MDS and AML patients with 1) > 20% blast cells with or without clonal chromosomal abnormality, 2) with complex chromosomal abnormalities, and 3) with simple and balanced rearrangements or a normal karyotyope but suspected cryptic abnormalities. This cytogenomic approach may not only provide a better diagnostic scheme to delineate breakpoints and gene contents of chromosomal and cryptic abnormalities in patients with MDS and AML, but hopefully allow identification of disease-causing or modifying candidate genes, and eventually lead to improved prognostification and treatment of patients with MDS and AML.

## Competing interests

The authors declare that they have no competing interests.

## Authors' contributions

PL was the principal investigator for this project and wrote the manuscript. PL, RB and FX coordinated the sample processing, data analysis, and literature review. PL, RB, FX and BX carried out aCGH analysis. KW, AJD and RK performed chromosome and FISH analyses. FX and AP participated in the BAC clone labeling and FISH analysis. SH provided clinical information and was involved in the discussions. All authors have read and approved the final manuscript.

## Supplementary Material

Additional file 1 **Table S1**. Segemental copy number alterations detected in the 30 MDS/AML patients. Click here for file

## References

[B1] Swerdlow SH, Campo E, Harris NL, Jaffe ES, Pileri SA, Stein H, Thiele J, Vardiman JW WHO classification of tumors of haematopoietic and lymphoid tissues 2008 International Agency for Research on Cancer (IARC)

[B2] Lowenberg B Downing JR Burnett A Acute myeloid leukemia New England J Med 1999 341 1051 1061 10.1056/NEJM199909303411407 10502596

[B3] Vance GH Kim H Hicks GA Cherry AM Higgins R Hulshizer RL Tallman MS Fernandex HF Dewald GW Utility of interphase FISH to stratify patient into cytogenetic risk categories at diagnosis of AML in an Eastern Cooperative Oncology Group (ECOG) clinical trial (E1900) Leukmia Res 2007 31 605 609 10.1016/j.leukres.2006.07.026 16996130

[B4] Xiang B Li A Valentin D Novak N Zhao HY Li P Analytical and clinical validity of whole genome oligonucleotide array comparative genomic hybridization for pediatric patients with mental retardation and developmental delay Am J Med Genet 2008 146A 1942 1954 10.1002/ajmg.a.32411 18627053

[B5] Xiang B Zhu H Shen Y Nasir R Sobeih M Miller D Lu K Hu X Andersson H Narumanchi TC Wang Y Martinez JE Wu BL Li P Li M Chen TJ Fan YS Genome-wide oligonucleotide Array CGH for etiological diagnosis of mental retardation: A multi-center experience on 1,499 clinical cases J Mol Diagn 2010 12 204 212 10.2353/jmoldx.2010.090115 20093387 PMC2871727

[B6] Toriello HV Goldenberg P Evidence-based medicine and practice guidelines: application to genetics Am J Med Genet 2009 151C 235 240 10.1002/ajmg.c.30222 19621463

[B7] Paciorkowski AR Fang M Chromosomal microarray interpretation: what is a child neurologist to do? Ped Neurol 2009 41 392 398 10.1016/j.pediatrneurol.2009.05.003 19931159

[B8] Rücker FG Bullinger L Schwaenen C Lipka DB Wessendorf S Fröhling S Bentz M Miller S Scholl C Schlenk RF Radlwimmer B Kestler HA Pollack JR Lichter P Döhner K Döhner H Disclosure of candidate genes in acute myeloid leukemia with complex karyotypes using microarray-based molecular characterization J Clin Oncol 2006 24 3887 3894 16864856 10.1200/JCO.2005.04.5450

[B9] Suela J Alvarez S Cifuenes F Largo C Ferreira BI Blesa D Ardan M Garcia R Marquea JA Odero MD Calasanz MJ Cigudosa JC DNA profiling analysis of 100 consecutive de novo acute myeloid leukemia cases reveals patterns of genomic instability that affect all cytogenetic risk groups Leukemia 2007 21 1124 1231 10.1038/sj.leu.2404653 17377590

[B10] Tyybäkinoja A Elonen E Piippo K Knuutila S Oligonucleotide array-CGH reveals cryptic gene copy number alterations in karyotypically normal acute myeloid leukemia Leukemia 2007 21 571 574 17268525 10.1038/sj.leu.2404543

[B11] Akagi T Ogawa S Dugas M Kawamata N Yamamoto G Nannya Y Sanada M Miller CW Yung A Schnittger S Haferlach T Haferlach C Koeffler HP Frequent genomic abnormalities in acute myeloid leukemia/myelodysplastic syndrome with normal karyotype Haematologica 2009 94 213 223 10.3324/haematol.13024 19144660 PMC2635399

[B12] Starczynowski DT Vercauteren S Telenius A Sung S Tohyama K Brooks-Wilson A Spinelli JJ Eaves CJ Eaves AC Horsman DE Lam WL Karsan A High-resolution whole genome tiling path array CGH analysis of CD34+cells from patients with low-risk myelodysplastic syndromes reveals cryptic copy number alterations and predicts overall and leukemia-free survival Blood 2009 112 3412 3424 10.1182/blood-2007-11-122028 18663149

[B13] Walter MJ Payton JE Ries RE Shannon WD Deshmukh H Zhao Y Baty J Heath S Westervelt P Watson MA Tomasson MH Nagarajan R O'Gara BP Bloomfield CD Mrózek K Selzer RR Richmond TA Kitzman J Geoghegan J Eis PS Maupin R Fulton RS McLellan M Wilson RK Mardis ER Link DC Graubert TA DiPersio JF Ley TJ Acquired copy number alterations in adult acute myeloid leukemia genomes Proc Natl Acad Sci USA 2009 106 12950 12955 10.1073/pnas.0903091106 19651600 PMC2716381

[B14] Radtke I Mullighan CG Ishii M Su X Cheng J Ma J Ganti R Cai Z Goorha S Pounds SB Cao X Obert C Armstrong J Zhang J Song G Ribeiro RC Rubnitz JE Raimondi SC Shurtleff SA Downing JR Genomic analysis reveals few genetic alterations in pediatric acute myeloid leukemia Proc Natl Acad Sci USA 2009 106 12944 12949 10.1073/pnas.0903142106 19651601 PMC2716382

[B15] Slovak ML Smith DD Bedell V Hsu YH O'Donnell M Forman SJ Gaal K McDaniel L Schultz R Ballif BC Shaffer LG Assessing karyotype precison by microarray-based comparative genomic hybridization in the myelodysplastic/myeloproliferative syndromes Mol Cytogenet 2010 3 23 10.1186/1755-8166-3-23 21078186 PMC3000833

[B16] Kamath A Tara H Xiang B Bajaj R He W Li P Double minute MYC amplification and deletion of MTAP, CDKN2A, CDKN2B and ELAVL2 in a patient with acute myeloid leukemia characterized by oligonucleotide-array comparative genomic hybridization Cancer Genet Cytogenet 2008 183 117 120 10.1016/j.cancergencyto.2008.02.011 18503831

[B17] Greenberg P Cox C LeBeau MM Fenaux P Morel P Sanz G Sanz M Vallespi T Hamblin T Oscier D Ohyashiki K Toyama K Aul C Mufti G Bennett J International scoring system for evaluating prognosis in myelodysplastic syndromes Blood 1997 89 2079 2088 9058730

[B18] Malcovati L Germing U Kuendgen A Della Porta MG Pascutto C Invernizzi R Giagounidis A Hildebrandt B Bernasconi P Knipp S Strupp C Lazzarino M Aul C Cazzola M Time-dependent prognostic scoring system for predicting survival and leukemic evolution in myelodysplastic syndromes J Clin Oncol 2007 25 3503 3510 10.1200/JCO.2006.08.5696 17687155

[B19] Haferlach C Bacher U Tiu R Maciejewski JP List A Myelodysplastic syndromes with del(5q): indications and strategies for cytogenetic testing Cancer Genet Cytogenet 2008 187 101 111 10.1016/j.cancergencyto.2008.08.002 19027491

[B20] Evers C Beier M Hildebrandt B Servan K Drechsler M Germing U Yoyer HD Royer-Pokora B Molcular definition of chromosome arm 5q deletion end points and detection of hidden aberrations in patients with myelodysplastic syndromes and isolated del(5q) using oligonucleotide array CGH Genes Chr Cancer 2007 46 1119 1128 10.1002/gcc.20498 17823930

[B21] Ebert BL Pretz J Bosco J Chang CY Tamayo P Galili N Raza A Root DE Attar E Ellis SR Golub TR Identification of RPS14 as a 5q-syndrome gene by RNA interference screen Nature 2008 451 335 339 10.1038/nature06494 18202658 PMC3771855

[B22] Graubert TA Payton MA Shao J Walgren RA Monahan RS Frater JL Walshauser MA Martin MG Kasai Y Walter MJ Integrated genomic analysis implicates haploinsufficiency of multiple chromosome 5q31.2 genes in de novo myelodysplastic syndromes pathogenesis PLoS One 2009 4 2 e4583 10.1371/journal.pone.0004583 19240791 PMC2642994

[B23] Starczynowski DT Kuchenbauer F Argiropoulos B Sung S Morin R Muranyi A Hirst M Hogge D Marra M Wells RA Buckstein R Lam W Humphries RK Karsan A Identification of miR-145 and miR-146a as mediators of the 5q- syndrome phenotype Nat Med 2010 16 49 58 10.1038/nm.2054 19898489

[B24] Starczynowski DT Karsan A Deregulation of innate immune signaling in myelodysplastic syndromes is associated with deletion of chromosome arm 5q Cell Cycle 2010 9 855 856 10.4161/cc.9.5.11156 20160505

[B25] Tchinda J Dijkhuizen T Vlies PvP Kok K Horst J Translocations involving 6p22 in acute myeloid leukaemia at relapse: breakpoint characterization using microarray-based comparative genomic hybridization Br J Haematol 2004 126 495 500 10.1111/j.1365-2141.2004.05082.x 15287941

[B26] Asou H Matsui H Ozaki Y Nagamachi A Nakamura M Aki D Inaba T Identification of a common microdeletion cluster in 7q21.3 subband among patients with myeloid leukemia and myelodysplastic syndrome Biochem Biophys Res Commun 2009 383 245 251 10.1016/j.bbrc.2009.04.004 19358830

[B27] Tyybäkinoja A Saarinen-Pihkala U Elonen E Knuutila S Amplified, lost, and fused genes in 11q23-25 amplicon in acute myeloid leukemia, an array-CGH study Genes Chr Cancer 2006 45 257 264 10.1002/gcc.20288 16283618

[B28] Zatkova A Merk S Wendehack M Bilban M Muzik EM Muradyan A Haferlach C Haferlach T Wimmer K Fonatsch C Ullmann R AML/MDS with 11q/MLL amplification show characteristic gene expression signature and interplay of DNA copy number changes Genes Chr Cancer 2009 48 510 520 10.1002/gcc.20658 19306356

[B29] Dolan M Peterson B Hirsch B Array-based comparative genomic hybridization characterizes a deletion associated with a t(15;17) in acute promyelocytic leukemia. Am J Clin Pathol 2008 130 818 23 18854276 10.1309/AJCPENMUI47OGKRW

[B30] Akagi T Shih LY Kato M Kawamata N Yamamoto G Sanada M Okamoto R Miller CW Liang DC Ogawa S Koeffler HP Hidden abnormalities and novel classification of t(15;17) acute promyelocytic leukemia (APL) based on genomic alterations Blood 2009 113 1741 1748 10.1182/blood-2007-12-130260 19109227 PMC2647673

[B31] Haferlach C Dicker F Kohlmann A Schindela S Weiss T Kern T Schnittger S Haferlach T AML with CBFB-MYH11 rearrangement demonstrate RAS pathway alterations in 92% of all cases including a high frequency of NF1 deletions Leukemia 2010 24 1065 1069 10.1038/leu.2010.22 20164853

[B32] Pasmant E Sabbagh A Masliah-Planchon J Haddad V Hamel MJ Laurendeau I Wolkenstein P Bieche I Vidaud M Vidaud D Detection and characterization of NF1 microdeletions by custom high resolution array CGH J Mol Diagn 2009 11 524 529 10.2353/jmoldx.2009.090064 19767589 PMC2765750

